# Decoding molecular distributional codes through collective instabilities

**Published:** 2026-07-22

**Authors:** Mason N. Rouches, Dongyang Li, Michael B. Elowitz, Arvind Murugan

**Affiliations:** 1 James Franck Institute, University of Chicago, Chicago, IL 60637; 2 Division of Biology and Biological Engineering, California Institute of Technology, Pasadena, CA 91125; 3 Howard Hughes Medical Institute

## Abstract

Biological information is often encoded in molecular variants that differ in just a few chemical traits, such as the number of phosphorylated sites or ubiquitin chain length, rather than in arbitrarily distinct species. These molecular distributions carry information about cellular state, yet reading them with conventional molecular circuits requires prohibitively many distinct sensors. In contrast, we show that collective physical instabilities can naturally integrate the information encoded in such distributions. Using an information-theoretic matching condition between an encoded distribution and a physical readout, we derive a geometric condition that any good decoder must satisfy, and establish that phase separation, percolation, and membrane curvature instabilities all approach it for biologically natural distributions while simple mass-action binding does not. Using mean-field theory and lattice Monte Carlo simulations, we find that phase separation reads the shape of a distribution beyond its mean, robustly capturing its variance and, more weakly, its skewness, whereas mass-action binding detects only the mean. Near phase boundaries the readout captures nearly all the information present in the molecular population. Finite valency, through the threshold for network formation, adds discriminatory power invisible to mean-field theory. These results suggest that cells can exploit collective physical instabilities as natural, compact, yet near-optimal sensors for decoding molecular distributional codes.

Biological information is often stored in molecular identity, i.e., in which molecules are present and in what amounts. To use that information, downstream processes must discriminate among molecules well enough to route them into distinct actions. When molecular classes are cleanly separable, a small set of sensors can reliably map composition to an appropriate response.

However, cellular information is not always carried by distinct, arbitrarily different species. Instead, it is often encoded in families of closely related molecular variants that differ only along a few chemical traits. In this case, a cellular condition then corresponds to a composition profile across that trait: the abundances P(n) of molecules with trait value n (e.g., the number and pattern of phosphorylations). Two environmental or cellular conditions may produce two overlapping distributions $P_{1}(\text{n})$ and $P_{2}(\text{n})$ that are difficult to distinguish. Such low-dimensional variation is widespread [1]: phosphorylation populates multiple modified forms of the same protein [2–4], ubiquitination generates families of chain lengths and linkage types [5, 6], glycosylation diversifies around a limited motif set [7, 8], many interaction domains span restricted families of binding preferences [9], and immune cell receptors interact with diverse pools of antigen. Biologically relevant signals are therefore often encoded in how a population of molecules is distributed across related variants that differ along a low-dimensional trait, rather than in the identity of any single form. We will refer to such encodings as distributional codes.

How are such distributional codes read out? In principle, a chemical reaction network can extract any feature of P(n), for instance by combining variant-specific binders or modifiers whose outputs are integrated downstream. But such circuits must be tailored to the feature of interest, with a wiring that grows with the size of the trait space.

An alternative is to use a single collective physical process whose macroscopic response is natively sensitive to the shape of a population; such processes can couple many weak molecular interactions into a single macroscopic readout without component-by-component tabulation. Phase separation is the most-studied example in cell biology [10], but related phenomena include polymer assembly, conformational spread and percolation in interaction networks [11, 12], and curvature-driven membrane remodeling [13]. In each case the transition condition itself depends on the shape P(n) of the underlying population, suggesting that collective transitions broadly are well suited to decoding distributional codes.

Here we formulate sensing as an information-theoretic matching problem between a population encoder P(n;λ) and a physical decoder. Three classes of collective transitions (phase separation, percolation, and membrane remodeling) satisfy the matching condition for biologically relevant distributions, while simple mass-action binding does not. We then focus on phase separation, where biological prevalence motivates a deeper treatment. We characterize the fidelity and robustness of phase-separation-based decoding using mean-field theory and use lattice Monte Carlo simulations to show that finite valency further expands its discriminatory capacity. These results identify collective transitions as a natural physical substrate for reading distributional codes.

## Optimizing Information transmission by matching Encoder-Decoder pairs —

**FIG. 1.**
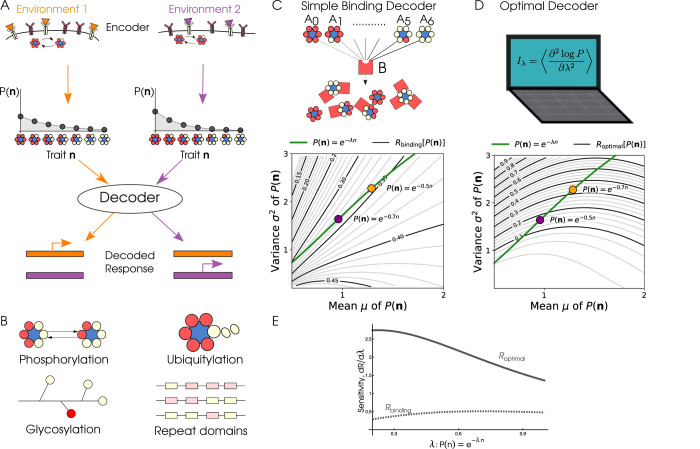
Decoding molecular distributional codes — (A) Environmental information is encoded into a distribution P(n;λ) over a molecular trait n by an upstream process (e.g., a signaling network), and decoded into a response, such as expression of a set of genes. A good decoder maps different environmental conditions (orange, purple) onto different responses. (B) Biological examples of low-dimensional traits: phosphorylation state, ubiquitin chain linkage, and glycosylation level or the number of repeats of a protein domain (C) (top) Simple binding model: a monovalent binder B interacts with molecules $A_{1},A_{2},\ldots ,A_{n}$ carrying 1,2,…,n binding sites respectively, with abundances set by P(n); the response $R_{\text{binding}}[P(n)]$ is the bound fraction of A. (bottom) An encoder that produces the exponential family $P(n)\propto e^{-\lambda n}$ traces a curve (green) in the $\left(\mu ,\sigma^{2}\right)$ plane as λ varies. Contours of $R_{\text{binding}}$ run nearly parallel to this encoder curve, so two representative distributions P(n) (orange, purple dots) yield nearly the same response. (D) An optimal decoder, $R_{\text{opt}}[P(n)]=\sum_{n}P(n)e^{\alpha n}$ (SI), of the same molecules $A_{n}$. Contours of $R_{\text{opt}}$ run nearly perpendicular to the encoder curve, maximizing the difference in response between the orange and purple distributions. (E) Sensitivity dR/dλ of response R to changes in λ of the input distribution $P(n)\propto e^{-\lambda n}.R_{\text{opt}}$ consistently outperforms $R_{\text{binding}}$.

We formalize collective decoding as an information transmission problem. An environmental parameter λ is encoded in a distribution P(n;λ) across a low-dimensional trait n, and a decoder maps this distribution to a physical output R[P(n)] such as the expression of a set of target genes (Fig. 1A). The Fisher information in R about λ is,

$$I_{\lambda }=\frac{1}{\sigma_{R}^{2}}{\left(\frac{\delta R}{\delta P(\text{n})}\cdot \frac{\partial P(\text{n})}{\partial \lambda }\right)}^{2}$$

where $\sigma_{R}^{2}$ is the noise in the decoder. The integrand factorizes into a decoder response sensitivity δR/δP(n) and an encoder direction ∂P(n)/∂λ, giving a geometric matching criterion (see Supplement): contours of constant response R must be orthogonal to the curve traced by P(n;λ) as λ varies. Instead, if R’s contours align with this curve, distinct environments collapse onto the same output and information is lost.

**FIG. 2.**
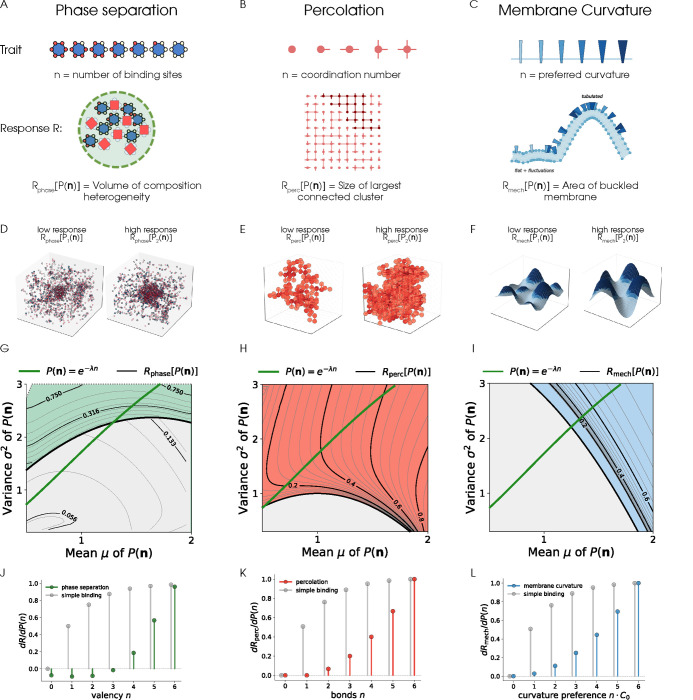
Response contours for collective instabilities are naturally suited for decoding exponential distributions — We consider three collective physical processes, each involving molecules $A_{n}$ that vary along a discrete trait n and with abundances P(n). (A - C): Traits (upper) and responses (lower) for the three physical systems. (A) **Phase-Separation**: The trait n is the number of binding sites for multivalent sticker molecule B. The response $R_{\text{phase}}$ is compositional heterogeneity. (B) **Percolation**: The trait n is the coordination number of sites on a cubic lattice. Response $R_{\text{perc}}$ is the number of sites in the largest cluster. (C) **Membrane Curvature**: The trait n is the preferred curvature of a membrane protein. The response $R_{\text{mech}}$ is the area of membrane that buckles. (D - F): Example configurations for two distributions $P_{1}(\text{n})$, $P_{2}(\text{n})$ (from numerical simulations). In each case, $P_{1}(\text{n})$ generates a low response, while $P_{2}(\text{n})$ generates a large response. (G – I) Contours of macroscopic response R (gray) are plotted in the ($\mu ,\sigma^{2}$) plane of input distributions P(n), alongside the curve (green) traced by the exponential family $P(n)\propto e^{-\lambda n}$. Response contours are nearly perpendicular to the encoded input distribution in each case. (J – L) Sensitivity of response R to components of the input distribution P(n). Sensitivity curve for collective decoders (colored) is convex (as required for near-optimal decoding of exponentials) while the independent binding model curve (gray) is concave.

**FIG. 3.**
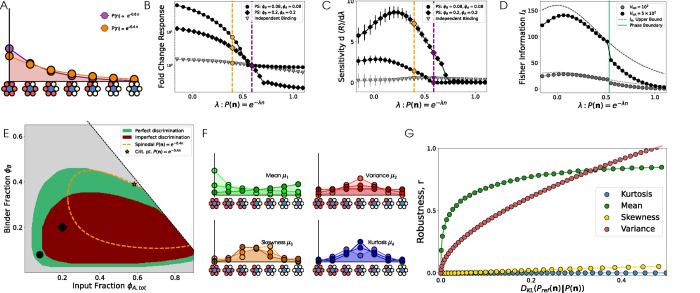
Fidelity & Robustness of Discrimination with Phase Separation — (A) Two input distributions P(n). (B) Response $R_{\text{phase}}$ to distributions $P(n)=e^{-\lambda n}$ parameterized by rate λ. Black diamonds and circles are the response of phase-separation at different system parameters. Gray triangles are the response $R_{\text{bind}}$ of mass action binding. Responses are normalized to the response to $P(n)=e^{-0.6n}$ (purple in A). (C) Sensitivity of the response versus distribution. Error bars denote intrinsic noise in the response, $Var\left(R_{\text{phase}}\right)$. Parameters same as in B. (D) Fisher information $I_{\lambda }$ versus λ, for several system sizes. Near the phase boundary (green line) the information nearly saturates the total information in the system. (E) Discrimination regimes in hyperparameter space $\left(\phi_{B},\phi_{A,\text{tot}}\right)$, the volume fractions of binder B and of all forms of A put together. (Green) *Perfect Discrimination*: only the orange input distribution in panel (A) phase-separates, distinguishable by physical state alone. (Red) *Imperfect Discrimination*: both inputs in (A) phase-separate, distinguishable by droplet volume. Circular and diamond points mark the hyperparameters ($\phi_{B},\phi_{A,\text{tot}}$) used in B-D. Dashed line, spinodal of $P(n)=e^{-0.4n}$; star is the critical point. (F) Maximum-entropy distribution families matched below moment order i and varying at i and above. Fixed moments $\mu_{1}=3$, $\mu_{2}=1.6$, $\mu_{3}=0$. (G) Hyperparameter space robustness r= Perfect/(Perfect + Imperfect) (fractional area of green region in (E)) versus distributional distance $D_{KL}\left(P_{\text{ref}}\|P\right)$, for the families in F.

We make these ideas concrete with a one-parameter family of encoders: exponential distributions $P(n)\propto e^{-\lambda n}$ over a discrete trait n such as phosphorylation number, ubiquitin chain length, or binding-domain repeat count (Fig. 1B). Exponentials are both mechanistically natural and biologically widespread. They arise whenever each step of a sequential modification reaction has a roughly constant per-step efficiency, so that the abundance of n-fold modified species falls geometrically with n. Such profiles are observed in polyubiquitin chain length distributions, sequential multisite phosphorylation, and template-free polymer/oligomer length distributions [5, 14–16] (an exponential distribution is also the maximum-entropy distribution over n given only its mean, making it the generic steady-state form when no further structure is imposed). We use this family as an illustrative case for Figs. 1–2; in Fig. 3 we relax it and ask how phase separation discriminates more general P(n), including families matched in mean and variance that differ only in higher moments such as skewness and kurtosis.

We first consider a minimal decoder: a binder molecule B that interacts independently with sites on the input molecules $A_{n}$ (Fig. 1C); the response $R_{\text{binding}}$ is the fraction of A molecules bound at least one B molecule. We plot contours of $R_{\text{binding}}$ in the ($\mu ,\sigma^{2}$) plane of the input distribution. For the exponential family $P(n)\propto e^{-\lambda n}$, the mean and variance satisfy $\sigma^{2}=\mu (\mu +1)$, so the encoder traces a near-parabola in the ($\mu ,\sigma^{2}$) plane that runs nearly tangent to the binding contours. By our criterion, simple binding is a poor decoder of exponential distributions: changes in λ move the encoded distribution along, rather than across, the decoder’s contours.

From our geometric picture, good decoders of the exponential family should have response contours where variance *decreases* with the mean, Figure 1D. As shown in the SI, such good decoders must weight high-n species more than linearly with n and in fact, the optimal decoder has exponential weighting, i.e., $R_{\text{opt}}[P(n)]=\sum_{n}P(n)e^{\alpha n}$ (Fig. 1D). Comparing sensitivities dR/dλ across the exponential family confirms that $R_{\text{opt}}$ outperforms simple binding for all λ, with the gap widening as the encoder curve becomes more parallel to the binding contours (Fig. 1E).

## Collective Instabilities decode exponential distributions —

What physical systems naturally approach the performance of the optimal decoder?

We extend the binding model by letting the binder B be multivalent (Fig. 2A). The encoded input is still a distribution P(n) of molecules $A_{n}$ carrying n sticker sites, but each binder B now exposes multiple binding sites and can thus bridge several $A_{n}$’s. This system is described by the physics of phase separation with a free energy:

$$\frac{F}{Vk_{B}T}=\sum_{n=0}^{N_{s}}\phi_{A,n}log\phi_{A,n}+\frac{nJ_{B}}{k_{B}T}\phi_{A,n}\phi_{B}\;\;\;\;+\phi_{B}log\phi_{B}+\left(1-\phi_{B}-\phi_{A,\text{tot}}\right)log\left(1-\phi_{B}-\phi_{A,\text{tot}}\right)$$

where $\phi_{A,n}$ is the volume fraction of the encoded input molecule $A_{n}$, $\phi_{B}$ is the volume fraction of the multivalent binder B, and $J_{B}$ is the interaction energy between a sticker site on $A_{n}$ and any site on B. The equilibrium state of the system is determined by minima of F. We define the response $R_{\text{phase}}[P(n)]$ as the volume of the dense phase enriched in A and B molecules, if it exists. When phases do not coexist, we define $R_{\text{phase}}[P(n)]$ as the correlation volume, or typical length scale of compositional fluctuations.

In Figure 2G we plot response contours of the phase-separating system in the plane of distributions P(n) parameterized by mean μ and variance $\sigma^{2}$. The phase-separation response $R_{\text{phase}}[P(n)]$ cuts across the encoding contour for exponential distributions $P(n)\propto e^{-\lambda n}$ nearly orthogonally.

Another broadly relevant collective physical process that is sensitive to a low-dimensional trait distribution is percolation, Figure 2B. Here the trait value sets the number (0,...,6) of available bonds per site on a cubic lattice, and the response $R_{\text{perc}}[P(n)]$ is taken to be the size of the largest connected cluster. The probability of adding a bond to a cluster depends only on the mean of the bond distribution, $p_{\text{bond}}\propto \mu$, while the statistics of cluster sizes depend on the entire distribution (see SI). For instance, the percolation threshold traces the curve $\sigma^{2}=2\mu -\mu^{2}$ in the space of distributions. Mean-field theory and Monte Carlo simulations show that contours of $R_{\text{perc}}[P(n)]$ are aligned to exponential distributions in the $\mu -\sigma^{2}$ plane, Figure 2H. The decoder’s sensitivity to the distribution, $\delta R_{\text{perc}}[P(n)]/\delta P(n)$ is convex in the bond number n, Fig. 2K, confirming that percolation is matched to exponential distributions.

Mechanical systems are also collective decoders. We consider an example of membrane bending by a family of curvature-generating proteins. Here we take a series of individual proteins to favor spontaneous membrane curvatures between 0 (flat) and $6c_{0}$ (highly curved), where $c_{0}$ is an intrinsic preference, Figure 2C. Each protein locally bends the membrane towards its preferred curvature. Crucially, highly curved regions preferentially recruit proteins favoring that curvature, leading to inhomogeneous curvature throughout the membrane – some regions might go unstable and buckle while others remain flat. The resulting equilibrium state of the membrane balances these curvature preferences, protein mixing entropy $f_{\text{mix}}(\vec{\phi })$, and tension-associated costs $\sigma_{\text{mem}}$ [17] (see Supplement for our detailed model). The free energy $F_{\text{mem}}$ of a membrane with mean curvature C and protein distribution $\vec{\phi }$ is

(1) $$F_{mem}[C,\vec{\phi }]=\int dS\left[\sigma_{mem}+\frac{\kappa }{2}{\left(C-C_{0}(\vec{\phi })\right)}^{2}+f_{mix}(\vec{\phi })\right]$$

where $C_{0}(\vec{\phi })\equiv c_{0,\text{mem}}+\sum_{n}c_{0}n\phi_{n}(\text{r})$ is the protein-modified spontaneous curvature preference, and κ the curvature modulus. We define the response $R_{\text{mech}}[P(n)]$ to be the fraction of buckled membrane, which is determined by minimization of $F_{\text{mem}}$ over protein densities and membrane shape (see Supplement). Again, these contours are nearly orthogonal in the $\mu -\sigma^{2}$ plane, Fig. 2I, and the decoder’s sensitivity is convex in the trait value, Fig. 2L.

All three phenomena here exploit *collective instabilities* that naturally couple to the full shape of the trait distribution P(n), not merely its mean. While a bespoke molecular circuit can be constructed to sense each trait value P(n) individually and integrate them in just the right way, these collective instabilities perform that integration for free, yielding response contours that are nearly optimal in an information-theoretic sense.

## Fidelity and Robustness of a phase-separation based decoder —

The appeal of collective decoding is its simplicity but it requires the system to sit near a collective instability. Consider two distinct environments encoded in the distributions of Fig. 3A. To distinguish these two distributions, the decoder’s response to one distribution must be much larger than it is for the other. For some system parameters, phase-separation based decoders perform well, while for others this amplification is poor, Fig. 3B. While evolution can plausibly tune parameters to keep a biological system near such an instability, this mode of sensing is broadly relevant only if the instability is accessible across a wide region of parameter space, not just at a fine-tuned point.

We now ask how precisely phase-separation based decoding can distinguish two input distributions P(n) at fixed hyperparameters given intrinsic noise (*fidelity*), and how large a region of hyperparameter space supports such discrimination (*robustness*). The relevant hyperparameters are the interaction strength $J_{B}$ and the binder and input volume fractions $\phi_{B},\phi_{A,\text{tot}}$. We first hold these hyperparameters fixed and characterize fidelity, then sweep over them to characterize robustness.

At a fixed hyperparameter choice, the intrinsic noise in the response R sets a precision floor on estimating λ, captured by the Fisher information $I_{\text{phase}}$. Combined with the noise floor on the input molecules, $I_{A}=\sigma^{2}\phi_{A,\text{tot}}V_{\text{tot}}$, the Fisher information on λ is $I_{\lambda }=\frac{I_{A}I_{\text{phase}}}{I_{A}+I_{\text{phase}}}$. As λ varies, the system passes through three regimes with different information content (Fig. 3C,D).

When input distributions do not drive phase separation, the response is small and the noise is on the same scale Fig. 3C. Here the information captured by phase-separation is small and a fraction of the total information available Fig. 3D. At values that do drive phase separation, the response is large with moderate noise, and phase-separation captures a large fraction of the available information. Near the threshold λ at which the system phase-separates, the response is large and the noise is small. Here phase-separation conveys nearly all of the available information, Fig 3D (green line).

No decoder can exceed the information limit set by counting individual molecules. What phase-separation can do is leverage the information present in larger systems by aggregating more molecules into a collective measurement: in most of parameter space the Fisher Information scales with system size, $I_{\text{phase}}∼V_{\text{tot}}$. Near critical points and first-order phase-boundaries, however, this scaling is greater than linear (see Supplement). Since $I_{\text{phase}}$ increases with system size, phase-separation can integrate most of the available information into a collective measurement, and near a phase-boundary can approach the limit set by molecular counting.

The Cramér-Rao bound, $Var(\delta \lambda )\geq 1/I_{\lambda }$, implies that $I_{\lambda }\approx 10^{2}$ can constrain an estimate of λ±0.1, sufficient to discriminate the two distributions in Fig. 3A (separated by $\lambda_{1}-\lambda_{2}\approx 0.2$), but only near the phase boundary.

Turning to robustness, we now ask how large a region of hyperparameter space supports reasonable fidelity at all. Overlaying the equilibrium phase diagrams of the two input distributions in the ($\phi_{B},\phi_{A,\text{tot}}$) plane (Fig. 3E) defines three regions. Where only one distribution phase-separates, the two are distinguishable by physical state alone and amplification can be made arbitrarily large; we call this *Perfect Discrimination*. Where both phase-separate, discrimination requires comparing droplet volumes and amplification saturates at the system scale; we call this *Imperfect Discrimination*. Outside both, only weak dilute-phase amplification is available, except near critical points. For the two example distributions in Fig. 3A, the Perfect and Imperfect zones together occupy a substantial fraction of the ($\phi_{B},\phi_{A,\text{tot}}$) plane. Discrimination is therefore accessible across a broad swath of hyperparameter space rather than only at a fine-tuned point.

Next we quantify the size of these zones in hyperparameter space $\left(\phi_{B},\phi_{A,\text{tot}}\right)$ plane as a measure of robustness. We define robustness by r= Perfect/(Perfect + Imperfect), plotted against the Kullback-Leibler divergence $D_{KL}(P(n)\|Q(n))$ between the two distributions (Fig. 3G). For distributions that differ only by mean or only by variance, the robustness r increases quickly as the input distributions become more distinguishable (i.e., higher $\left.D_{KL}(P(n)\|Q(n))\right)$. Hence, our mean-field theory description of phase separation predicts robust discrimination of input distributions over a large region of hyperparameters.

We then asked whether phase separation can discriminate distributions that share the same mean and variance but differ in higher-order moments such as skewness or kurtosis. To pose these tasks, we generated families of maximum-entropy distributions with matched lower moments and varying higher ones (Fig. 3F). The robustness curves for skewness and kurtosis saturate at very low values (Fig. 3G), indicating that these tasks remain difficult for phase separation even when the distributions are highly distinguishable. This asymmetry between low and high moments has an analytical origin in the structure of the phase boundary. The spinodal condition (Supplement),

(2) $$\frac{\phi_{B}\left(1-\phi_{B}\right)\left(\sigma^{2}+\mu^{2}\left(1-\phi_{A,\text{tot}}\right)\right)\phi_{A,\text{tot}}}{{\left(\phi_{A,\text{tot}}\mu \phi_{B}+k_{B}T/J_{B}\right)}^{2}}=1,$$

depends on μ and $\sigma^{2}$ but not on higher moments of P(n), while critical-point conditions add a dependence on the skewness $\mu_{3}$. Phase diagrams of distributions that differ at the level of kurtosis thus have identical critical points and spinodals, limiting a large region of parameter space to imperfect discrimination. This implies a vanishing bound on robustness for kurtosis and higher moments, and a non-zero bound for mean, variance, and skewness discrimination.

These results treat binders and inputs as independent. Finite valency can saturate available binding sites in ways that may expand or constrain the discriminable region, which we address next with lattice Monte-Carlo simulations.

## Discriminating molecular distributions with Monte-Carlo simulations —

Mean-field theory weights each molecule $A_{n}$’s contribution to phase separation by its sticker count n, but misses a key aspect of phase separation: molecules with n≤2 cannot drive phase separation since network assembly requires n≳3 [18, 19]. We therefore expect finite valency to *enhance* discriminatory capacity beyond the mean-field bounds of Fig. 3G, because, e.g., distributions P(n) matched in mean but differing in mass above the bridging threshold n=3 should give qualitatively different responses.

To test this, we performed lattice Monte-Carlo simulations of an explicit-valency analogue of the Fig. 2A model. Each input molecule $A_{n}$ carries exactly n binder-binding sites with n∈{0,…,6} and each binder B carries $n_{B}=4$ sites; particles occupy a cubic lattice and exchange under particle-swap and bond-rearrangement moves accepted with a Metropolis criterion (see Supplement for details). The readout $R_{MC}[P(\text{n})]$ is a linear classifier on the binned cluster-size distribution, with weights fit to separate the two target distributions.

**FIG. 4.**
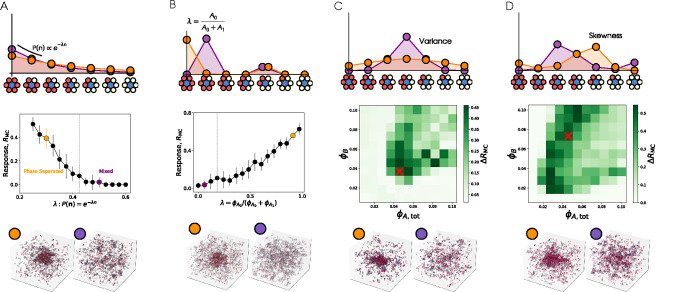
Finite valency effects, captured through Monte-Carlo simulations, enhance discrimination — (A) We used Monte-Carlo simulations to discriminate the (i) two exponential distributions shown. (ii) Response $R_{MC}[P(\text{n})]$ is the output of a linear classifier on the binned cluster size distribution. Dashed line is the estimated phase-boundary. (iii) Representative configurations from simulations of the two distributions. (B) Same analysis as in (A) but to discriminate distributions with the same multivalent content ($\phi_{A_{4}}$) and differ entirely in the amount of singly-valent complexes $\lambda =\frac{\phi_{A_{0}}}{\phi_{A_{1}+\phi_{A_{0}}}}$. Distributions with high amounts of singly-valent molecules sequester binders, inhibiting phase-separation. (C) Robustness analysis for (i) input distributions with equal mean binding sites and different variance. (ii) Discrimination between the two distributions as a function of hyperparameters $\phi_{B},\phi_{A,\text{tot}}$. Color is a proxy for fidelity and indicates the difference in response between the two distributions, $\Delta R_{MC}=R_{MC}\left[P_{1}(\text{n})\right]-R_{MC}\left[P_{2}(\text{n})\right]$. Darker points are more distinguishable than lighter points. (D) Same analysis as in (C) for input distributions that differ in skewness alone but equal mean and variance.

For exponential inputs, $P(n)\propto e^{-\lambda n}$, at fixed hyperparameters, the readout switches sharply with λ across the simulated phase boundary (Fig. 4A), reproducing the mean-field picture of Fig. 1D. The bridging threshold then produces a discrimination axis invisible to mean-field theory. Mixing $A_{0}$, $A_{1}$, and $A_{4}$ at fixed multivalent content $\phi_{A_{4}}$ while replacing $A_{0}$ with $A_{1}$ raises the mean sticker count, which should promote phase separation in the mean-field picture; in simulation it does the opposite (Fig. 4B), because each $A_{1}$ caps a site on the binder without contributing to bridging. The same trait shift moves the mean-field and lattice responses in opposite directions. Finally, we find that discrimination of higher moments is more robust than mean-field predicts. To quantify this we mapped the response $R_{MC}$ across hyperparameters $\left(\phi_{B},\phi_{A,\text{tot}}\right)$ for distributions that differ in variance (Fig. 4C) and skewness (Fig. 4D). Both pairs admit broad discriminable regions of high fidelity where the differential response, $\Delta R_{MC}=R_{MC}\left(P_{1}\right)-R_{MC}\left(P_{2}\right)$, is large. Skewness is not visibly harder than variance, partially lifting the mean-field bound of Fig. 3G.

## Discussion —

Our central result is that collective physical instabilities are natural decoders of molecular distributional information. Information theory predicts a geometric matching condition, namely that efficient decoders must have response contours crossing the encoder trajectory orthogonally. Phase separation, percolation, and membrane curvature remodeling all satisfy this condition for the encoder families studied here, while simple mass-action binding does not (Figs. 1–2). For phase separation specifically, this sensitivity can be understood analytically; the mean-field spinodal (Eq. 2) is already sensitive to μ and $\sigma^{2}$, even before accounting for the basic network-formation requirement of n≥3 binding sites needed for phase separation. Lattice Monte Carlo simulations incorporating finite valency expand this further, yielding sensitivity to $\mu_{3}$. Both model classes are equilibrium; non-equilibrium drive remains an important open extension.

Compared to simple binding networks, such instability-based decoding is more compact in that it collapses sensing and response into a single physical act. For example, consider how proteins marked for destruction through ubiquitination are sensed and destroyed (by autophagy or the proteasome) [20, 21]. Ubiquitin ligases polymerize chains of ubiquitin on proteins, earmarking them for degradation. Polyubiquitinated proteins promote the condensation of specific (autophagocytic) degradatory machinery, while monoubiquitinated proteins do not [21, 22]. In this way sensing (condensation) and actuation (substrate selection and degradation) are a concerted physical process.

Our results extend prior work in two directions. Studies of valency effects on phase boundaries have focused on monodisperse systems [23]; we show that the distribution of valencies can qualitatively reshape phase diagrams. The broader multicomponent phase-separation literature has largely considered molecules with statistically random and distinct interactions [24–26], modeling a regime where individual species are highly dissimilar. We study the opposite limit, of molecules that are nearly identical, differing only along a low-dimensional trait. Polydispersity theory [27, 28] established that phase behavior depends on the full composition of a mixture; we add an information-theoretic lens that asks which distributional features are discriminable and at what cost in decoder parameter tuning.

Distributional codes are inexpensive to generate. Template-free modification processes (polyubiquitination, multisite phosphorylation) produce statistical profiles across molecular variants without requiring enzymatic specificity for each form [29, 30], and the resulting signal is robust since it is a statistical property of a population rather than the precise state of any one molecule. Structured sequential modification processes such as kinetic proofreading also generate exponential distributions over modification states as a natural byproduct, [29, 31], placing these encoders precisely in the class for which collective instabilities serve as natural decoders.

A concrete example occurs in the discrimination of self from non-self ligand by the T-cell receptor (TCR). The statistics of ligand (un)binding events drive a concert of phosphorylations that culminates in the multi-site phosphorylation of order hundred transmembrane LAT molecules [32, 33]. Phosphorylated LAT phase-separates on the plasma membrane to form large signaling complexes [34], that polymerize actin and can lead to T-cell activation. Here the TCR encodes binding statistics into a distribution over LAT phosphorylation states. This distribution is decoded by phase-separation, which combines the noisy measurement and weak interactions in every LAT molecule into an informative, macroscopic readout.

In B-cell affinity maturation, collective physical readouts of antigen affinity can be more informative than molecular level readouts [35, 36]. B-cells are selected on the basis of their ability to bind to specific antigen, though activation itself could be sensitive to antigen distributions.

Our work suggests several natural extensions. Cooperative binding, internal linker flexibility [37, 38], and multi-component condensates could expand discriminatory capacity, and the encoder-decoder matching framework provides a principled criterion for evaluating each. Phase morphologies may further serve as readouts for sequential reactions at condensate interfaces [39]. More broadly, these results suggest a rationale for why biological information is so often carried by distributions over related molecular variants rather than by arbitrarily distinct species. Both the encoding (imprecise modification) and the decoding (collective assembly near a thermodynamic instability) exploit processes that are physically natural at the molecular scale, rather than demanding fine-tuned specificity at either stage.

## Supplementary Material

Supplement 1
